# Efficiency of Various Tubular Occlusion Agents in Human Dentin after In-Office Tooth Bleaching

**DOI:** 10.3390/jfb14080430

**Published:** 2023-08-17

**Authors:** Natalia Papazisi, Dimitrios Dionysopoulos, Olga Naka, Dimitris Strakas, Sotiria Davidopoulou, Kosmas Tolidis

**Affiliations:** 1 Department of Operative Dentistry, School of Dentistry, Aristotle University of Thessaloniki, 54124 Thessaloniki, Greece; natalia.papazisi@gmail.com (N.P.); dimitris.strakas@gmail.com (D.S.); sdavidop@dent.auth.gr (S.D.); ktolidis@dent.auth.gr (K.T.); 2 Department of Prosthodontics, School of Dentistry, Aristotle University of Thessaloniki, 54124 Thessaloniki, Greece; naka@dent.auth.gr

**Keywords:** bioactive glass, dentin hypersensitivity, Er,Cr:YSGG laser, occlusion of dentinal tubules, stannous fluoride

## Abstract

The aim of this laboratory study was to investigate and compare the impact of five desensitizing techniques as a treatment fortooth sensitivity on the exposed dentin after an in-office tooth bleaching procedure. Thirty intact human molars were collected for this investigation. The specimens were obtained by transversely cutting 2.5 mm of the crowns, leading to exposure of the dentin. The specimens were cleaned in an ultrasonic bath and treated initially with EDTA gel 15% for 4 min and then with Opalescence Boost bleaching gel (40% H_2_O_2_) for two sets of 20 min. Then, the samples were randomly divided into six groups (*n* = 5) and received one of the following treatments: Group 1 (no treatment—control group), Group 2 (Emofluor gel—0.4% SnF_2_), Group 3 (MI Paste—CPP-ACPF), Group 4 (BioMinF paste—calcium phospho-fluoro-silicate), Group 5 (air-abrasion with ProSylc—Bioglass 45S5), and Group 6 (Er,Cr:YSGG laser). Subsequently, each sample was observed utilizing scanning electron microscopy (SEM) in order to detect the rate of occlusion of dentin tubules. SEM-EDS analysis revealed no occlusion of the dentin tubules in the control group, while Groups 2, 4, and 5 presented high effectiveness (>95% percentage of occluded tubules), and Groups 3 and 6 presented lower values (21.6 and 26.8%, respectively). It was concluded that althoughall the tested groups presented higher percentages of occlusion of the dentinal tubules compared to the control group, there were differences in effectiveness among them. The most effective treatments were the daily use of BioMinF paste and SnF_2_-containing gel, as well as air-abrasion with ProSylc powder.

## 1. Introduction

Dentin hypersensitivity, also known as tooth sensitivity, is a common dental condition that affects a significant number of people worldwide. It is characterized by acute, temporary pain or discomfort experienced when the teeth are exposed to certain stimuli such as hot or cold temperatures, sweet or acidic foods, or even simple actions like brushing or flossing [1]. This condition may considerably affect an individual’s quality of life, causing a modification of eating habits, avoidance of certain foods or drinks, and sensitivity-related anxiety or discomfort during oral care routines [2].

Dentin is the porous tissue beneath the hard outer layer of enamel and cementum, and it contains microscopic tubules that connect to the tooth’s nerve endings. When the protective layers of enamel or cementum are eroded or worn away, the tubules become exposed, allowing external stimuli to reach the nerves within the tooth, leading to the characteristic sharp pain or discomfort [3].

Tooth bleaching, which involves the application of peroxide-based products to whiten teeth, can sometimes lead to temporary or prolonged sensitivity in the teeth. This sensitivity occurs when the bleaching agents penetrate the enamel and reach the dentin or when they are accidentally applied directly tothe exposed dentin [4]. It is important to note that this sensitivity is generally temporary and should subside over time. However, if the sensitivity persists or worsens, it is advisable to consult a dentist who can recommend appropriate treatments or desensitizing products to alleviate the discomfort and restore pulpal health [5].

The main mechanism to achieve a desensitizing effect is to block the exposed dentinal tubules leading to interruption of the external stimulation [6]. Thankfully, there are various treatment options available for occluding dentinal tubules and addressing dentin hypersensitivity after tooth bleaching. These treatments include the daily use of desensitizing toothpaste and gels containing stannous fluoride or/and sodium fluoride [7], casein phosphopeptide–amorphous calcium phosphate (CPP-ACP) [8], and bioactive glass [9]. Additionally, in some cases, in-office techniques should be used, such as air-abrasion with bioactive materials [10] or the application of laser irradiation [11].

Therefore, the purpose of the current research was to evaluate and compare the effects of five desensitizing techniques as a treatment fortooth sensitivity on the exposed dentin after an in-office tooth bleaching procedure. The tested therapies included (a) three daily (for 14 days) treatments using products thatcontain stannous fluoride (SnF_2_), casein phosphopeptide–amorphous calcium phosphate with sodium fluoride (CPP-ACPF), and calcium phospho-fluoro-silicate glass, and (b) two in-office treatments of air-abrasion with Bioglass 45S5 and Er,Cr:YSGG laser irradiation. SEM-EDS analysis was used to investigate the rate of occlusion of the exposed dentin tubules.

Two null hypotheses were set prior to the study. H_0_1: The tested treatments would induce the same rate of occlusion of dentin tubules asthe control group, where no treatment was received. H_0_2: The tested treatments would exhibit the same effectiveness regarding the occlusion of the dentin tubules.

## 2. Materials and Methods

### 2.1. Preparation of Specimens

**Figure 1:**
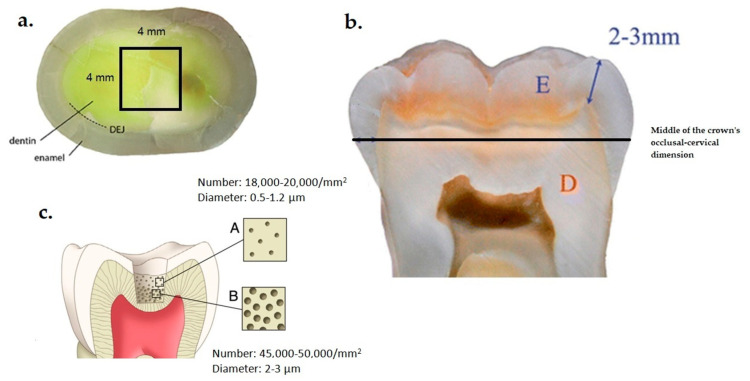
(**a**) The dentin area of the specimens that was evaluated; (**b**) the exact position of the cut of the teeth aiming to expose the dentin (E: enamel, and D: dentin); (**c**) the density and the diameter of the exposed dentinal tubules depending on the depth of the cut (A: occlusal dentin part, and B: medium dentin part).

The local Ethical and Research Committee (No. 145/11-03-2022) approved this study, which was conducted in accordance with the ethical standards outlined in the 1964 Declaration of Helsinki and its subsequent amendments, while adhering to the policies of the Aristotle University of Thessaloniki. A total of thirty healthy human molars were extracted due to periodontal reasons and stored in a 0.5% chloramine T solution at 6 °C for up to 3 months. The age range of the patients included in this study was 5 years, and they provided their informed consent for the use of their teeth forresearch purposes. Following the extraction of the teeth, any remaining soft tissues were meticulously eliminated, and the tooth surfaces were cleansed using a mixture of pumice and water. The teeth were then placed in acrylic resin until they were positioned approximately 2 mm below the cement–enamel junction. To expose the dentin surface (Figure 1a), the enamel was precisely cut at the middle of the crown’s occlusal–cervical dimension (Figure 1b) using a low-speed precision sectioning machine (Isomet 11-1180 Buehler, Lake Bluff, IL, USA) with water cooling. At this depth of dentin, the number of the exposed tubules varies from 18,000–20,000/mm^2^ to 45,000–50,000/mm^2^, and the diameter of the tubules from 0.5–1.2 μm to 2–3 μm, depending on the cutting depth (Figure 1c). The cut surface of each tooth specimen was ground on a polishing machine (Jean Wirtz TG 250, Dusseldorf, Germany) with 200 rpm under water cooling (50 mL/min) using gradually 600-, 800-, and 1000-grit silicon carbide abrasive papers (Apex S system, Buehler, Lake Bluff, IL, USA) for 20 s each. Subsequently, the dentin specimens were treated with 15% EDTA (Endo-Prep Cream, CERKAMED, Stalowa Wola, Poland) for 4 min to eliminate the smear layer on the dentin tubule orifices, and then rinsed with distilled water and air-dried. Then, the specimens were immersed in an ultrasonic bath (Euronda Spa, Montecchio Precalcino, Vicenza, Italy) for 5 min to remove the residual smear layer and were stored in artificial saliva at 37 ± 1 °C until used. The composition of the artificial saliva was 0.103 g/L CaCl_2_, 0.019 g/L MgCl_2_·6H_2_O, 0.544 g/L KH_2_PO_4_, and 2.24 g/LKCl, and buffer (TCP-KOH) was added to adjust the pH to 7 [12].

### 2.2. Tooth Bleaching Procedure

The cut surface of the tooth specimens underwent an in-office bleaching treatment using a red-colored bleaching agent with a concentration of 40% H_2_O_2_ (Opalescence Boost PF 40, Ultradent Products, Inc., South Jordan, UT, USA). The bleaching agent was mixed by combining the contents of two syringes, and this mixture was applied following the manufacturer’s instructions. A layer of approximately 1 mm thickness was evenly spread across the entire surface of the specimens. The gel was left on the surface for 20 min, with agitation occurring every 5 min. Afterward, the gel was removed from the dentin surface using a high-power dry suction. This procedure was repeated for a total of 40 min, and after the second application, the specimens were rinsed with distilled water. Finally, all specimens were stored in artificial saliva at 37 ± 1 °C.

### 2.3. Experimental Groups of the Study

Following the bleaching procedure, the tooth specimens were randomly divided into 6 groups (*n* = 5) and received one of the following desensitizing protocols:

Group 1 (control group): No treatment was received.

Group 2: The specimens were smeared daily for two weeks with Emofluor^®^ gel (Dr. Wild & Co. AG, Muttenz, Switzerland), which contains 0.4% stannous fluoride (SnF_2_), and the gel remained undisturbed for 1 min on dentin. Then, the samples were rinsed with distilled water and stored in artificial saliva at 37 ± 1 °C.

Group 3: The specimens were smeared daily for two weeks with GC MI Paste Plus (GC Corp., Tokyo, Japan), which contains casein phosphopeptide–amorphous calcium phosphate (CPP-ACPF) and 900 ppmF^−^ for 3 min, and then rinsed and stored as in Group 2.

Group 4: The specimens were smeared daily for two weeks with BioMinF paste (Cera Dynamics Ltd., Fenton, UK), which contains a fluoride-containing BAG (calcium phospho-fluoro-silicate) for 3 min, and then rinsed and stored as in Group 2.

Groups 5: The dentin surfaces underwent treatment using an Aquacare™ clinical air-abrasion unit (Velopex, Harlesden, UK). The samples were treated with ProSylc powder (Velopex, Harlesden, UK), which includes BAG 45S5 (NovaMin^®^, Alachua, FL, USA). Each specimen underwent a 10 s sandblasting pretreatment in wet mode, where the air stream was shielded by a curtain of de-ionized water. The operational settings were adjusted as follows: air pressure at 20 psi (approximately 1.38 bar), powder flow rate dial set to 1 g/min, nozzle angle at 90°, nozzle–surface distance at 5 mm, and internal nozzle diameter of 900 μm [13]. Air-abrasion was performed only once on the initial experimental day and then stored in artificial saliva at 37 ± 1 °C.

Group 6: An Er,Cr:YSGG (2780 nm) solid-state laser system (Waterlase MD Turbo, BIOLASE, Irvine, CA, USA) was used to irradiate each specimen. The laser beam had a divergence of α_total_ = 14° and followed a Gaussian energy distribution profile. The pulse repetition rate was set at 20 Hz with no water flow (0%) and 10% airflow, and the pulse duration remained constant at 140 μs. A Z-type glass cylindrical tip (MZ6) with a diameter of 600 μm and a length of 6 mm was attached to a gold handpiece of the laser system. The tip was positioned 1 mm away from the dentin surface in the focused mode. To ensure consistent spot size during hand irradiation, an endodontic file was fixed to the handpiece and maintained a distance of 1 mm from the surface during irradiation. Each pulse delivered energy of 12.5 mJ, resulting in a peak power of 89.28 W. The handpiece was placed perpendicular to the dentin surface, and the specimens were irradiated by hand in a scanning mode. The handpiece was moving slowly both horizontally and vertically at a speed of 2 mm/s to achieve uniform irradiation and cover the entire specimen area (16 mm^2^). This procedure was carried out for all specimens by the same operator. The total irradiation time was 20 s (10 s vertically and 10 s horizontally), and the average output power was 0.25 W. This corresponded to a power density of 88.46 W/cm^2^ and a fluence of 4.42 J/cm^2^. The laser parameters were adjusted mainly based on the work of Yilmaz and Bayindir [11]. Laser treatment was also performed only once on the initial experimental day and then stored in artificial saliva at 37 ± 1 °C.

The technical characteristics of the products investigated in this study are presented in Table 1.

### 2.4. Evaluation of Occlusion of Dentin Tubules Using SEM-EDS Analysis

Scanning electron microscopy (JEOL Ltd., JSM-840, Tokyo, Japan) was used to examine the impact of the tested treatments on the occlusion of dentin tubules. The observations of dentin were implemented in the center of the cut surface and within a square area of 4 mm × 4 mm (Figure 1a). The specimens were mounted on aluminum stubs, sputter-coated with carbon to a thickness of about 200 Å in a vacuum evaporator at low vacuum, and observed at an accelerated voltage of 20 kV. Ten SEM photomicrographs were taken from different areas of dentin surfaces at ×500 magnifications to observe changes in surface morphology. Moreover, another ten images were performed at ×1000 magnification to evaluate the percent of the dentin tubules (%) that were occluded and at ×3000 magnification to measure the level of occlusion and the diameter of the tubules after the treatments.

**Figure 2:**
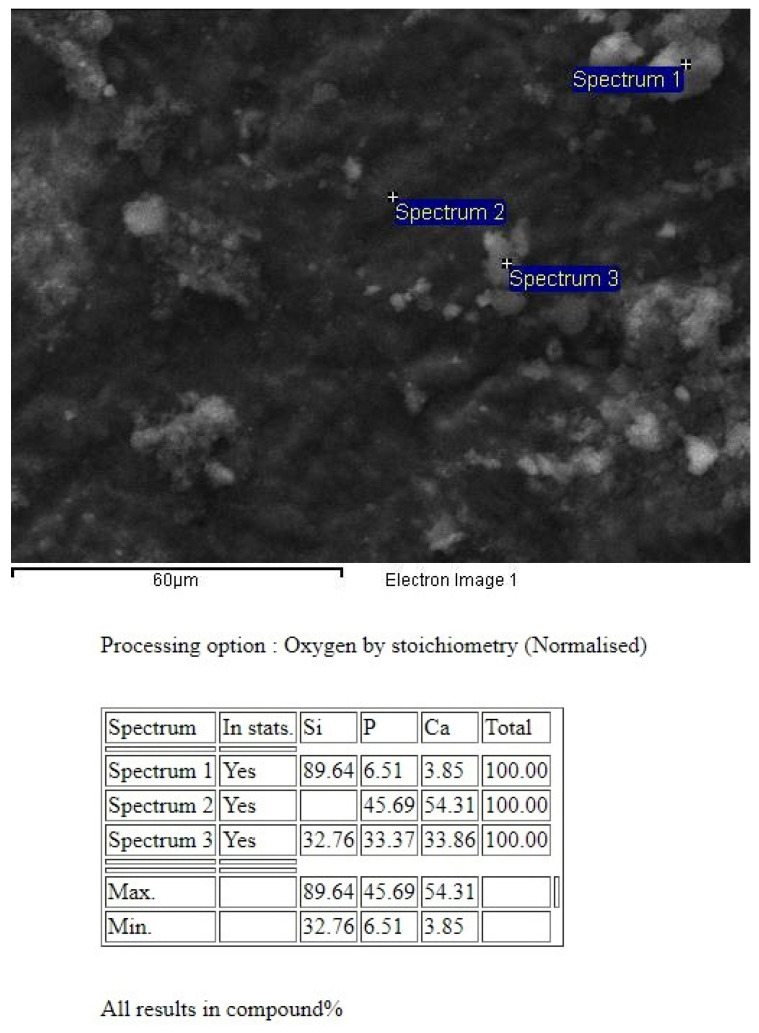
Representative SEM image identifying particles and precipitations that occluded the dentin tubules utilizing energy-dispersive X-ray spectroscopy.

The images were assessed for the measurement of tubule occlusion by two independent blinded evaluators and the scores were averaged. Energy-dispersive X-ray spectroscopy (EDS) was also applied to identify the nature of occluding particles in the tubules (Figure 2).

### 2.5. Statistical Analysis

The statistical analysis of the data was implemented using IBM Corp’s SPSS Statistics 25.0 software (Chicago, IL, USA). The data’s normality and homogeneity were assessed through the Shapiro–Wilk and Levene tests, respectively. The diameter of open tubules (μm) and the number of open tubules per 0.01 μm^2^ were subjected to a one-way ANOVA, and significant differences between the experimental groups were revealed using Tukey’s test at a significance level of 0.05. As the evaluators provided tubule occlusion levels as scores, a nonparametric test was used for the evaluation procedure. The Kruskal–Wallis test was employed to compare these values among the different groups. Additionally, the Mann–Whitney test was used to make separate comparisons between the groups. Furthermore, a *p*-value of less than 0.05 indicated the presence of a significant difference between the tested groups.

## 3. Results

**Figure 3:**
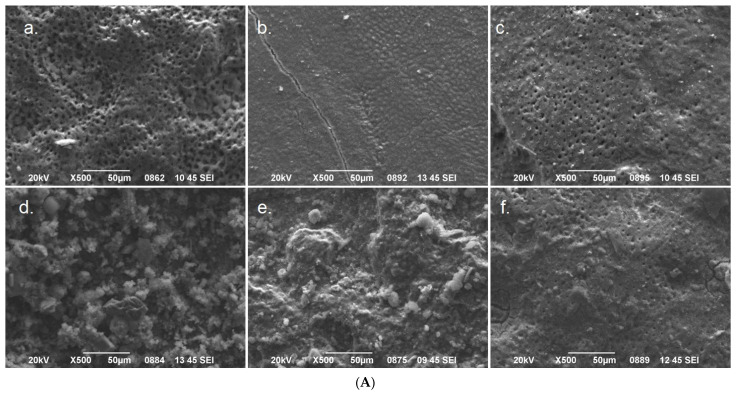

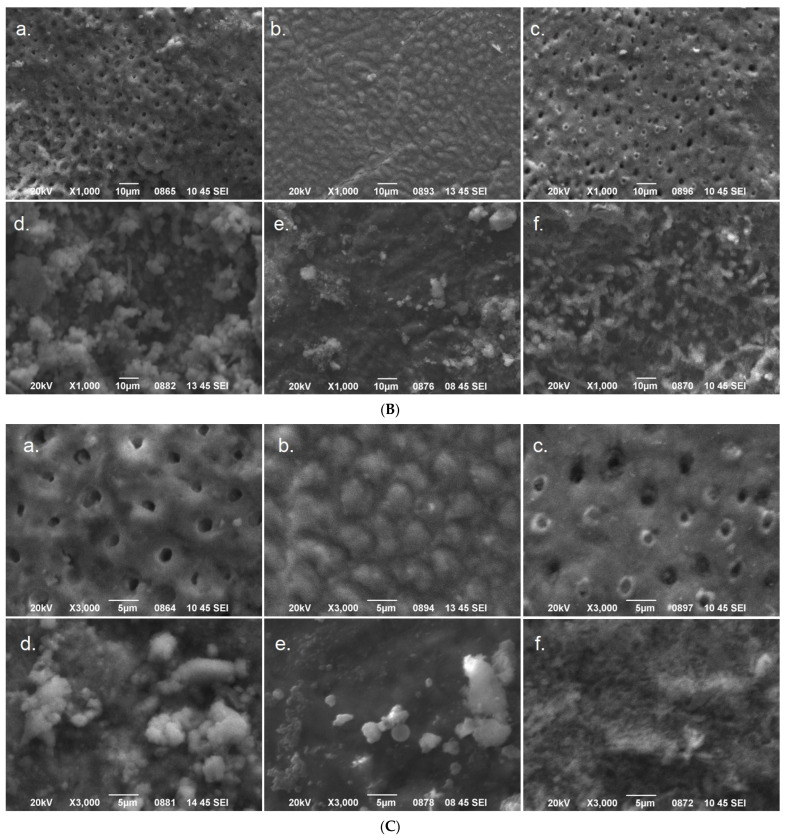
(**A**) Representative SEM photomicrographs showing the dentin surface after the treatments of each experimental group at ×500 magnification. (**a**) Control group; (**b**) SnF_2_ treatment; (**c**) CPP-ACPF treatment; (**d**) calcium phospho-fluoro-silicate glass treatment; (**e**) bioglass 45S5 treatment; (**f**) Er,Cr:YSGG laser treatment. (**B**) Representative SEM photomicrographs showing the dentin surface after the treatments of each experimental group at ×1000 magnification. (**a**) Control group; (**b**) SnF_2_ treatment; (**c**) CPP-ACPF treatment; (**d**) calcium phospho-fluoro-silicate glass treatment; (**e**) bioglass 45S5 treatment; (**f**) Er,Cr:YSGG laser treatment. (**C**) Representative SEM photomicrographs showing the dentin surface after the treatments of each experimental group at ×3000 magnification. (**a**) Control group; (**b**) SnF_2_ treatment; (**c**) CPP-ACPF treatment; (**d**) calcium phospho-fluoro-silicate glass treatment; (**e**) bioglass 45S5 treatment; (**f**) Er,Cr:YSGG laser treatment.

Means and standard deviations of the diameter of open tubules (μm), the level of tubule occlusion (scale 0–2), and the number of open tubules per 0.01 mm^2^ dentin surface of the experimental groups of the study are presented in Table 2. Figure 3A–C illustrates representative SEM images of the treated dentin surfaces of each experimental group at ×500, ×1000, and ×3000 magnification.

All treatments exhibited beneficial effects regarding occlusion of dentin tubules compared to the control group (*p* < 0.05). The most effective treatments were sandblasting with ProSylc and daily use of BioMinF paste and Emofluor gel (SnF_2_), which did not significantly differ from each other (*p* > 0.05) and occluded almost all dentin tubules (>99%), as it can be observed in Figure 3A–C. The use of Er,Cr:YSGG laser also was effective on dentin tubule occlusion but to a much lesser extent (26.8%), while CPP-ACPF paste application showed similar effectiveness tothe laser treatment (21.6%).

**Figure 4:**
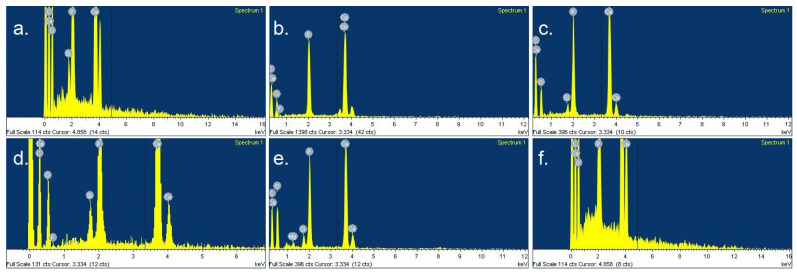
EDS spectra of the above representative SEM photomicrographs of each experimental group at ×3000 magnification. (**a**) Control group; (**b**) SnF_2_ treatment; (**c**) CPP-ACPF treatment; (**d**) calcium phospho-fluoro-silicate glass treatment; (**e**) bioglass 45S5 treatment; (**f**) Er,Cr:YSGG laser treatment.

The extent of occlusion of the dentin tubules was in accordance with the number of occluded tubules (Table 2). The average diameter of the dentin tubules was reduced after all the tested treatments in comparison with the untreated specimens (*p* < 0.05). This decrease in the diameter of the tubules was attributed to different mechanisms depending on the applied treatment. EDS analysis revealed the nature of the particles or precipitations that contributed to the occlusion of the tubules (Figure 4 and Table 3).

In particular, for air-abrasion treatments with bioactive glass particles, the occlusion of the tubules was achieved by a layer of hydroxyapatite that was formed on the dentin’s surface. The application of Emofluor, which contains SnF_2_, created a Sn-containing layer on the surface, while the application of GC MI Paste Plus occluded the tubules with calcium phosphate and calcium fluoride compounds. Finally, Er,Cr:YSGG laser irradiation reduced the diameter of the tubules presumably by partially melting the dentin.

## 4. Discussion

In spite of notable advancements in comprehending dentin hypersensitivity, a deficiency in clinical research supported by empirical evidence persists, particularly in relation to comprehending the underlying mechanisms of pain. Consequently, our grasp of this condition remains constrained. Currently, the most widely embraced theory is Brännström’s hydrodynamic theory [14]. According to this theory, the sensitivity of dentin is predominantly attributed to the movement of fluid within the dentinal tubules, triggered by external stimuli, which subsequently activates pain receptors situated in the interface region between the pulp and dentin [15]. It is postulated that intradental myelinated A-β fibers and certain A-δ fibers are accountable for responding to stimuli that lead to fluid displacement within the dentin tubules, thereby causing the characteristic brief, intense pain sensation [16]. This is why the majority of proposed therapies for tooth sensitivity aim to achieve the closure of dentin tubules, thus impeding the flow of dentinal fluid.

Post-vital tooth bleaching often gives rise to tooth sensitivity, which raises significant concerns as it affects a substantial portion of the population and tends to persist for an average duration of 1 to 4 days. This sensitivity is thought to arise when hydrogen peroxide molecules infiltrate the enamel and dentin, eventually reaching the pulp and triggering an inflammatory response [17]. It is crucial to acknowledge that this process diverges from the underlying causes of typical dentin hypersensitivity. Ordinarily, sensitivity provoked by bleaching is transitory in nature. However, if individuals with pre-existing dentin sensitivity undergo bleaching procedures, the resulting sensitivity can be notably intense and prolonged [16].

In the current research, the primary focus was to assess the efficacy of different therapies in addressing tooth sensitivity by occluding the dentinal tubules. To evaluate the effectiveness of each therapy, we compared the treated specimens with a control group that did not receive any treatment. The evaluation of dentin tubule occlusion was performed by conducting SEM-EDS analysis on representative images captured at different magnifications. This method of analysis has been previously employed in similar studies [7,11,18,19].

According to the outcomes of the current investigation, H_0_1, which stated that the tested treatments would induce the same occlusion of dentin tubules as the control group of the study, was rejected. Indeed, all the tested treatments induced occlusion of the dentinal tubules and presented a significantly higher number of occluded dentin tubules than the control group. Nevertheless, the mechanism of dentin tubule occlusion was different for each therapy. Moreover, there were significant differences in the extent of occlusion among the tested treatments, which means that H_0_2, which stated that the tested treatments would exhibit the same effectiveness regarding occlusion of the dentin tubules, was also rejected. The most effective methods in the present investigation were the use of stannous fluoride, calcium phosphο-fluoro-silicate, and bioglass 45S5. Yet, previous studies have yielded conflicting outcomes regarding the degree to which they are capable of obstructing dentin tubules [9,20].

Stannous fluoride has exhibited remarkable efficacy in the closure of dentin tubules, likely attributable to the potent affinity of Sn ions to the mineral surfaces of tooth hard tissues [21]. These findings are consistent with the conclusions drawn from the current investigation. This hypothesis posits that following an abrasive challenge like tooth bleaching, SnF2 forms a protective layer on the tooth surface, establishing bonds with exposed tissues. In particular, tin ions are incorporated into the near-surface layer of eroded tooth surfaces, penetrating to a depth of approximately 20 μm. These depositions offer hours of safeguarding against abrasive challenges, shielding the underlying surface from further erosive exposure. Additionally, research has indicated that the connection formed between F and Ca resulting from the application of various fluoride salts is less robust compared to the bond established on tooth surfaces treated with SnF2. The interaction between stabilized SnF2 and dentin is more intricate than a simple attachment between F and exposed Ca sites on the outer layer of the tooth [22]. Hines et al. [7] documented that following 8 weeks of utilizing a toothpaste containing 0.454% SnF2, the dentin surfaces displayed effective coating, with the patent dentin tubules being sealed. In comparison to the control toothpaste, the experimental toothpaste exhibited a noticeable reduction in tooth sensitivity, underscoring its significant effectiveness. However, despite its efficacy, SnF2 does present certain clinical limitations, including a metallic taste, the potential for tooth staining, and potential loss of taste sensation with prolonged usage [22].

Bioactive glasses stand as specialized biomaterials composed of glass, distinguished by their propensity for surface reactivity. When these glasses come into contact with tooth surfaces and interact with saliva under conditions of acidity, they possess the capability to dissolve, thereby liberating calcium and phosphate ions while concurrently elevating the pH [23,24]. This sequence of events initiates a cascade of chemical responses, culminating in the creation of a layer composed of hydroxycarbonate apatite. This apatite layer exhibits parallels to the mineral hydroxyapatite commonly present in dentin, and both substances can establish a chemical bond, thereby fostering a more robust attachment of the glass particles to the dentin surface [25]. It is pertinent to acknowledge that the full maturation of this apatite layer takes some time, often spanning several hours. This progressive development of reparative dentin, characterized by low permeability, impedes the diffusion of detrimental agents from dentin tubules toward the dental pulp. Consequently, it curtails the movement of dentin fluid, providing an enduring remedy for tooth sensitivity [9].

ProSylc integrates Bioglass 45S5, which is a calcium sodiumphospho-silicate compound commercially known as NovaMin^®^. Typically, glasses are non-crystalline solids lacking a definite crystal structure, predominantly composed of silica-based components with minor additions. However, Bioglass 45S5 deviates from this norm by featuring reduced levels of silica and heightened concentrations of calcium and phosphorus compared to typical glasses [26]. In the context of the current study, the utilization of ProSylc air-abrasion unveiled the presence of a safeguarding layer of hydroxyapatite, as evidenced by SEM-EDS analysis. This hydroxyapatite layer, enriched with calcium and phosphorus, encompassed nearly the entirety of the dentin surface. Furthermore, an additional peak corresponding to silicon (Si) was detected, signifying the inclusion of bioactive glass. These outcomes signify the successful integration of bioactive glass particles into the treated dentin, facilitating remineralization stimulation and closure of the dentinal tubules.

The mode of action of Bioglass 45S5 involves a localized and temporary increase in pH, which occurs as a result of the release of sodium ions (Na^+^). This pH increase triggers the precipitation of phosphate and calcium ions. As a consequence of this process, a layer of calcium phosphate is formed. These released ions and the resulting calcium phosphate layer are responsible for the occlusion of tubules during the treatment of tooth sensitivity [27].

BioMinF^®^ is a specially formulated calcium phospho-fluoro-silicate intended for use as an additive in toothpaste. It differs from Bioglass 45S5 by containing fluoride and having significantly higher phosphate content. The higher phosphate content is believed to accelerate apatite formation, leading to an increased amount of apatite being produced. This increase in phosphate also results in a considerable reduction in pH during dissolution [28]. The presence of small amounts of fluoride in the glass contributes to the rapid formation of apatite, favoring the creation of fluorapatite over hydroxycarbonated apatite. As the fluoride content in the glass is increased, the rise in pH is further diminished, potentially leading to the formation of CaF_2_ at the expense of fluoroapatite [29]. The formed layer of fluoroapatite is more resistant to erosive agents and thus may contribute to a longer occlusion of the dentin tubules.

The utilization of CPP-ACPF paste demonstrated a less robust effect compared to the previously mentioned treatments concerning the closure of dentin tubules. However, it displayed more favorable outcomes when contrasted with the control group. The mode of action for CPP-ACP involves multiple processes that contribute to its advantageous impacts on tooth surfaces. To begin with, CPP-ACP increases the number of potential binding sites on the tooth surface for calcium, leading to a reduction in the continuous diffusion of calcium. Additionally, ACP operates as a buffer for free calcium and phosphate ions present on dentin, assisting in maintaining a state of supersaturation. This state is pivotal as it mitigates demineralization and amplifies the remineralization of the tooth structure [30]. Another rationale for the positive consequences of CPP-ACP is that its application facilitates the development of a crystalline layer, which envelops the openings of dentin tubules. This protective layer serves as a shield against external stimuli, thereby affording extra safeguarding to the tooth surface [31]. Prior investigations have documented the formation of a safeguarding layer on the tooth surface subsequent to the utilization of CPP-ACP [32]. Nonetheless, this layer is not entirely uniform; it manifests adherent irregularities and appears as clusters of structures that fill the interprismatic cavities while partially covering the tubules. These findings align with the outcomes presented in the present study.

The application of the Er,Cr:YSGG laser resulted in the closure of dentin tubules, albeit to a lesser degree when compared to treatments involving bioactive glasses and stannous fluoride. The interaction between the Er,Cr:YSGG laser and dentin is subject to the influence of several irradiation parameters, encompassing pulse duration, average power, repetition rate, spot size, method of delivery, characteristics of the laser beam, as well as dentin’s optical properties encompassing refractive index, scattering coefficient, and absorption coefficient [33]. Operating at a wavelength of 2780 nm, the Er,Cr:YSGG laser exhibits a notably high affinity for water and hydroxyapatite, both prominent constituents of dentin [34]. This attribute leads to limited penetration of the laser beam into dentin, with the bulk of laser energy being absorbed within the surface layers of dentin. This specific trait offers an advantage by safeguarding the dental pulp from harm [35].

Various explanations exist regarding the impact of laser exposure on dentin, though the most widely embraced one proposes that it revolves around the closure or blockage of dentin tubules by means of dentin melting and subsequent recrystallization processes [36]. When tackling tooth sensitivity, it is imperative that the laser beam avoids eroding the treated dentin surface, instead triggering only structural and chemical transformations. The erosion of dentin leads to a surface devoid of a smear layer, open dentinal tubules, and uneven textures that lack demineralization—an undesirable outcome for the procedure [37]. Furthermore, the utilization of laser settings surpassing 0.75 W might result in the formation of charred spots, rendering such protocols unsuitable for addressing dentin hypersensitivity [38]. In response to these concerns, the present research adhered to a strategy where the Er,Cr:YSGG laser’s average power for dentin irradiation was maintained at a level below that causing erosion (0.25 W), following the guidelines outlined in a prior investigation [11].

Suggestions have been put forth indicating that employing lower power settings could initiate the dissipation of dentinal fluid. This, in turn, could lead to a decrease in dentin permeability, ultimately resulting in a reduction in dentinal pain [36]. Corroborating this notion, a previous investigation conducted by Gholami et al. [39] demonstrated the successful melting of peritubular dentin through the utilization of the Er,Cr:YSGG laser. This process either partially or fully obstructed dentinal tubules, subsequently providing relief from hypersensitivity symptoms in patients. These results align harmoniously with the scanning electron microscopy (SEM) observations presented in the current study.

Limitations of this study include the lack of correlation of the results with clinical symptoms and the absence of data on the duration of therapeutic outcomes. Furthermore, in clinical conditions, the exposed dentin is typically located in the cervical region of the teeth, and toothbrushing habits may also influence the progression and longevity of tooth sensitivity.

## 5. Conclusions

Within the limitations of this in vitro study, it can be concluded that the tested treatments using bioactive glasses and stannous fluoride were highly effective in occluding dentin tubules after an in-office tooth bleaching procedure. The use of the tested protocols of Er,Cr:YSGG laser irradiation and CPP-ACPF was less effective, but still showed better outcomes compared with the control group of the study. Therefore, these tested treatments for tooth sensitivity could be valuable in alleviating symptoms after tooth bleaching. However, further studies are necessary to confirm the results of the current study, particularly clinical trials that can validate their clinical significance.

## Figures and Tables

**Table 1:** The composition of the tested products of the study according to manufacturers.

Product	Type	Composition	Active Agents	Manufacturer
Emofluor^®^	gel	Aqua, glycerin, propylene glycol, PEG-40-hydrogenated castor oil, cellulose gum, PEG-8, phosphocolamine, aroma, 0.4% SnF_2_ (1000 ppmF^−^), sodium saccharin	Stannous fluoride (SnF_2_)	Dr. Wild & Co. AG, Muttenz, Switzerland
GC MI Paste Plus	paste	Pure water, glycerol, Recaldent (CPP-ACP), D-sorbitol, CMC-Na, propylene glycol, silicon and titanium dioxide, xylitol, phosphoric acid, flavor, sodium fluoride (900 ppmF^−^), sodium saccharin, ethyl-, propyl-, butyl-, p-hydroxybenzoate	Recaldent (CPP-ACP) + sodium fluoride (NaF)	GC Corp., Tokyo, Japan
BioMinF^®^	paste	Particle size: 60–100 μm, 36–40 mol% SiO_2_, 22–24 mol% Na_2_O, 28–30 mol% CaO, 4–6 mol% P_2_O_5_, 1.5–3.0 mol% CaF_2_	Bioactive glass (calcium phospho-fluoro-silicate)	Cera Dynamics Ltd. Fountain Street, Fenton, Stoke-on-Trent ST4 2HB, UK
ProSylc	powder	Water, glycerin, hydrated silica, PVM/MA copolymer, sodium lauryl sulfate, cellulose gum, aroma, sodium hydroxide, carrageenan, sodium fluoride (1450 ppmF^−^), triclosan, sodium saccharin, limonene, CI 77891	NovaMin^®^ (Bioglass 45S5)	Velopex, Harlesden, UK

**Table 2:** Means and standard deviations of the diameter of open tubules (μm), the level of tubule occlusion (scale 0–2), and the number of open tubules per 0.01 mm^2^ of the experimental groups of the study after the treatments. The percentage (%) of the occluded tubules over the total number of tubules visible in the images (×1000 magnification) of each experimental group is also presented.

Group (Active Agent)	Diameter of Open Tubules in μm	Level of Tubule Occlusion (Scale 0–2)	Number of Open Tubules per 0.01 mm^2^	Percentage of Occluded Tubules (%)
Group 1 (control)	2.2 ± 0.4 ^A^	0.2 ± 0.2 ^A^	203.4 ± 15.3 ^A^	2.6%
Group 2 (SnF_2_)	0.3 ± 0.1 ^B^	1.9 ± 0.1 ^B^	1.4 ± 0.3 ^B^	99.5%
Group 3 (CPP-ACPF)	1.4 ± 0.3 ^C^	0.8 ± 0.2 ^C^	176.8 ± 12.6 ^C^	21.6%
Group 4 (calcium phospho-fluoro-silicate)	0.1 ± 0.1 ^B^	1.9 ± 0.1 ^B^	1.0 ± 0.4 ^B^	99.8%
Group 5 (Bioglass 45S5)	0.1 ± 0.1 ^B^	1.9 ± 0.1 ^B^	1.1 ± 0.3 ^B^	99.7%
Group 6 (Er,Cr:YSGG laser)	1.0 ± 0.3 ^D^	1.3 ± 0.3 ^D^	150.6 ± 13.9 ^D^	26.8%

Same uppercase superscripts in columns indicate no significant differences among the treatments (*p* > 0.05).

**Table 3:** Means and standard deviations of elemental content (wt%) of dentin surface after tooth bleaching of each experimental group of the study.

Elements	Control	Emofluor	MI Paste	BioMinF	ProSylc	Er,Cr:YSGG Laser
Ca	36.76 ± 4.62 ^a^	30.70 ± 4.36 ^a^	35.35 ± 3.18 ^a^	35.99 ± 4.82 ^a^	34.05 ± 3.56 ^a^	36.03 ± 5.13 ^a^
P	20.11 ± 3.39 ^a^	19.80 ± 2.12 ^a^	20.65 ± 2.02 ^a^	18.15 ± 1.75 ^a^	20.02 ± 1.88 ^a^	21.64 ± 2.10 ^a^
Si	1.16 ± 0.45 ^a^	0.00 ± 0.00 ^b^	0.69 ± 0.24 ^a^	2.17 ± 0.61 ^c^	2.13 ± 1.03 ^c^	0.00 ± 0.00 ^b^
F	0.00 ± 0.00 ^a^	3.60 ± 1.40 ^b^	0.82 ± 0.23 ^c^	3.41 ± 1.11 ^b^	0.00 ± 0.00 ^a^	0.00 ± 0.00 ^a^
Sn	0.00 ± 0.00 ^a^	6.36 ± 2.33 ^b^	0.00 ± 0.00 ^a^	0.00 ± 0.00 ^a^	0.00 ± 0.00 ^a^	0.00 ± 0.00 ^a^
Mg	0.00 ± 0.00 ^a^	0.00 ± 0.00 ^a^	0.00 ± 0.00 ^a^	0.00 ± 0.00 ^a^	1.16 ± 0.31 ^b^	0.00 ± 0.00 ^a^
O	41.97 ± 6.86 ^a^	39.54 ± 4.77 ^a^	42.49 ± 5.27 ^a^	40.28 ± 5.09 ^a^	42.64 ± 7.24 ^a^	42.33 ± 6.42 ^a^

Different lowercase superscripts in rows indicate statistically significant difference (*p* < 0.05).

## Data Availability

The data presented in this study are available upon request from the corresponding author. The data are not publicly available due to privacy.
